# Curcumin C3 complex®/Bioperine® has antineoplastic activity in mesothelioma: an in vitro and in vivo analysis

**DOI:** 10.1186/s13046-019-1368-8

**Published:** 2019-08-16

**Authors:** Francesco Di Meo, Stefania Filosa, Michele Madonna, Gerarda Giello, Alba Di Pardo, Vittorio Maglione, Alfonso Baldi, Stefania Crispi

**Affiliations:** 1 Institute of Biosciences and BioResources-UOS Naples CNR, via P. Castellino, 111-, 80131 Naples, Italy; 2 Department of Biology, University of Naples Federico II, Complesso Universitario Monte Sant’Angelo via Cinthia, Naples, Italy; 3 IRCCS Neuromed, Localitá Camerelle, Pozzilli, IS Italy; 4 Department of Environmental, Biological and Pharmaceutical Sciences and Technologies, University of Campania “L. Vanvitelli”, Caserta, Italy

**Keywords:** Mesothelioma, Curcumin C3 complex, Intrinsic apoptosis, Tumor growth inhibition

## Abstract

**Background:**

A major limitation in the treatment for malignant mesothelioma is related to serious side effects caused by chemotherapeutics and to the development of cancer-resistance. Advances in cancer therapies have been reached thanks to the introduction of alternative approaches, such as the use of phytochemicals. Curcumin-C3complex®/Bioperine® is a commercially standardized extract containing a ratio-defined mixture of three curcuminoids and piperine that greatly increase its bioavailability. Interestingly, the anticancer effect of this formulation has been described in different studies and several clinical trials have been started, but to our knowledge none refers to human mesothelioma.

**Methods:**

Curcumin-C3complex®/Bioperine® anticancer effect was evaluated in vitro in different human mesothelioma cell lines analysing cell proliferation, colony-forming assay, wound healing assays, invasion assay and FACS analysis. In vivo anticancer properties were analysed in a mesothelioma xenograft mouse model in CD1 Nude mice.

**Results:**

Curcumin-C3complex®/Bioperine® in vitro induced growth inhibition in all mesothelioma cell lines analysed in a dose- and time-depended manner and reduced self-renewal cell migration and cell invasive ability. Cell death was due to apoptosis. The analysis of the molecular signalling pathway suggested that intrinsic apoptotic pathway is activated by this treatment. This treatment in vivo delayed the growth of the ectopic tumours in a mesothelioma xenograft mouse model.

**Conclusions:**

Curcumin-C3complex®/Bioperine® treatment strongly reduces in vitro tumorigenic properties of mesothelioma cells by impairing cellular self-renewal ability, proliferative cell rate and cell migration and delays tumor growth in xenograft mouse model by reducing angiogenesis and increasing apoptosis. Considering that curcumin in vivo synergizes drug effects, its administration to treatment regimen may help to enhance drug therapeutic efficacy in mesothelioma. Our results suggest that implementation of standard pharmacological therapies with novel compounds may pave the way to develop alternative approaches to mesothelioma.

**Electronic supplementary material:**

The online version of this article (10.1186/s13046-019-1368-8) contains supplementary material, which is available to authorized users.

## Background

Malignant Mesothelioma (MM) is a rare and aggressive form of cancer primarily associated with exposure to asbestos fibres that affects the mesothelium surface of the pleural cavity. Despite the rarity of this disease, MM incidence is increasing worldwide and it is estimated to peak over the next 15 years with an increase of 5.4% per year [1]. The prognosis of MM is very poor due to the long latency development (30–40 years), to the diagnosis at a very late stage and to its high chemo-resistance. To date, the standard therapeutic modalities for this type of cancer, including chemotherapy, surgery and radiotherapy have yielded unsatisfactory outcomes [2]. Thus, the development of alternative and more effective therapies is an urgent requirement.

Previously, our group has investigated the in vitro and in vivo efficacy of a piroxicam and cisplatin combined treatment in MM. This treatment determined a marked tumor growth inhibition and an extended survival both in mouse models [3] and in spontaneous MM in pets [4]. Despite the efficacy of this treatment, drug toxicity and tumor-resistance represent a serious limitation. For this reason, research is needed on novel therapeutic approaches using natural compounds with no or little cytotoxicity. In this perspective, phytochemicals represent good candidates to be used alone or associated with standard chemotherapy. Phytochemicals are bioactive plant compounds that display an adjuvant effect resulting in tumor growth inhibition and in chemoprotective action towards the healthy cells with no obvious associated side effects [5].

Among others, curcumin - normally found in the turmeric of *Curcuma longa* Linn - is a naturally occurring phytochemical that has been widely used for centuries for the treatment of several diseases [6]. The use of curcumin in cancer is based on its ability to block the proliferation of tumor cells. Curcumin modulates cell cycle regulatory proteins involved in the pathogenesis and the prognosis of several cancers, including mesothelioma [7]. More interestingly, curcumin seems to induce a selective cytotoxicity toward cancer cells blocking the expression of molecules involved in cancer growth, such as nuclear factor NFkB and thioredoxin reductase (TrxR) [8–10]. In addition, curcumin is able to overcome the multidrug resistance of cancer cells down-regulating proteins responsible for the high drug efflux in multi-drug-resistant cancer cells [11].

Increasing evidences point out a robust anti-cancer efficacy of curcumin, however more attention should be paid to the formulations used, since in most of the in vivo studies and clinical trials no-standardized curcuminoid mixtures have been used [6].

Despite its numerous applications, the pharmacological potential of curcumin is severely restricted due to its poor water solubility, photodegradation, chemical instability and rapid metabolism as well as to its poor systemic bioavailability after oral administration [12]. In order to take advantages of the beneficial effects that curcumin may have, numerous attempts have been made to increase its efficacy and bioavailability. To overcome solubility problems our group as well as others have previously investigated the bioactivity of curcumin formulations using nanocarriers for delivery and targeting. These studies indicated that curcumin efficacy is tightly linked to its bioavailability [13, 14].

Other strategies investigated the efficacy of curcumin in combination with various molecules. Among them, the most promising one is represented by the co-administration of curcumin with piperine, an alkaloid of black pepper and long pepper. Piperine significantly enhances curcumin bioavailability – up to 2000% - by preventing its metabolism through the inhibition of the glucuronidation processes [15, 16].

In this study we investigated the anticancer activity of a commercially available preparation of curcumin and piperine (C3 complex® and Bioperine®, Sabinsa Corporation, CBP). C3 complex is a standardized extract containing a ratio-defined mixture of three curcuminoids (curcumin, bisdemethoxycurcumin, and demethoxycurcumin) that recently achieved the GRAS (Generally Recognized as Safe) status.

The efficacy of CPB was evaluated in vitro in different human mesothelioma cell lines and in vivo using a xenograft mouse model of MM. Overall, our data highlighted that CBP induces apoptosis in a time-dependent manner in MM cell lines sensitive (MSTO-221H, NCI-H2452) and insensitive (Ist-Mes2) to the piroxicam and cisplatin treatment [17] and that it is able to reduce tumor growth in a mouse MM model.

## Methods

### Cell culture and chemicals

Human MM cell lines were maintained at 37 °C in a 5% CO_2_ humidified incubator in either RPMI-1640 medium (MSTO-221H, NCI-H2452) or Dulbecco’s Modified Eagle’s Medium (Ist-Mes-2) supplemented with 10% fetal bovine serum (FBS), glutamine (2 mM), sodium pyruvate and antibiotics (0.02 IU/mL-1 penicillin and 0.02 mg/mL-1 streptomycin).

Curcumin C3 complex® (C3) and Bioperine® (BP) were provided from Sabinsa (Sabinsa Corporation, NJ, USA). Curcumin and Bioperine (CBP) stock solution contains 20 mM C3 complex® and 26 nM of Bioperine® in DMSO. This means that in all the experiments C3 is added to BP in the ratio 100:1 in weight (100 g of C3 for 1 g of BP).

### In vitro uptake of curcumin C3 complex®

MSTO-221H cells were seeded at a density of 2 × 10^5^ cells/well in six-well plates, and exposed to C3 with the concentration of 20 μM for 10′. For nuclear counterstain Hoechst 33342 (Invitrogen, Thermofisher, Waltham, MA, USA) was added in the culture medium. Since curcumin exhibits autofluorescence when excited at 455 nm and emits at 540 nm [18], uptake of the molecule was monitored under fluorescent microscope (DMI8, Leica, Instruments, Germany) using GFP filter with 20x magnification.

### Cell proliferation assay

To evaluate the effect of curcumin or CBP on cell proliferation, approximately 1 × 10^4^ cells/well in 48-well plates were plated and treated with 5, 10 and 20 μM for 24, 48 or 72 h. Cells were fixed with 3.7% formaldehyde for 10 min, washed with PBS and stained with 0.5% crystal violet for 10 min. A microplate reader (Cytation3 ASHI, BioTek, VT, USA) was then used to measure the absorbance at 595 nm. All the experiments were performed in triplicate. Data are expressed as the mean ± SD.

### Colony formation assay

Cells were seeded at a density of 500 cells/well in six-well plates and incubated for 7 days. Then, cells were treated with 20 μM for 24 h before replacing the media. Cells were grown for additional 7 days and then colonies were stained with crystal violet and counted. Representative plates were captured using scanner (Epson Stylus Photo, PX 650). All the experiments were performed in triplicate. Data are expressed as the mean ± SD.

### Wound healing assays

For wound healing assay, approximately 3 × 10^4^ cells/well were plated in six-well plates. After overnight incubation, wounds were created using a 200 μl pipette tip. Cells were treated with 20 μM of CBP for 24, 48 or 72 h. Representative plates were photographed using phase contrast microscope (DMI8, Leica, Instruments, Germany). The gap was photographed and measured using Image J software. All the experiments were performed in triplicate. Data are expressed as the mean ± SD.

### Invasion assay

The ability of cells to invade into the matrix and migrate towards was analysed in vitro using 24-well inserts with a pore size of 8 μm (Falcon, Corning NY) coated with Matrigel (BD Biosciences, Franklin Lakes, NJ) according to the manufacturer’s guidelines. Approximately 2 × 10^4^ cells were seeded in 250 μL of serum free medium with 20 μM of CBP in the upper surface of chamber; the lower chamber was filled with 750 μL of medium with 10% FBS. After 24 h of treatment, non-invasive cells remaining in upper chamber were removed by PBS washing. Invasive cells that had penetrated the Matrigel (the lower surface) were fixed with 3.7% formaldehyde for 10 min, washed with methanol for 20 min and stained with 0.5% crystal violet for 10 min, and then counted. Cells that invaded the lower surface of the filters were surveyed under a microscope at 10× magnifications, and five fields were randomly selected. Representative plates were photographed using bright field microscope (DMI8, Leica, Instruments, Germany).

All the experiments were performed in triplicate. Data are expressed as the mean ± SD.

### FACS analysis

Approximately 7,5 × 10^5^ cells/well were plated in 100 mm plates. After overnight incubation, cells were treated with 20 μM of CBP for 24, 48 or 72 h and stained with propidium iodide and annexin V (BD Biosciences, Franklin Lakes, NJ), according to the manufacturer’s protocols. Flow cytometry was performed using a FACSCanto TM flow cytometry system (Becton Dickinson, San Jose, CA). All the experiments were performed in triplicate. Data are expressed as the mean ± SD.

### RNA extraction and q-PCR

RNA from treated or untreated cells was extracted using Trizol (Thermo Fisher Scientific, MA USA) following manufacturer’s instructions. 200 ng of total RNA from each sample were retro-transcribed using the High Capacity cDNA Reverse Transcription Kit (Applied Biosystem, Thermo Fisher Scientific, MA USA). qPCR reactions were performed by means of a 7900 HT Real Time PCR (Applied Biosystem) using gene specific primers for the following selected genes:

BAX: Forward 5′-TTTGCTTCAGGGTTTCATCCA-3′: Reverse 5′- CTCCATGTTACTGTCCAGTTCGT-3′; BCL-2: Forward 5′-GTTCCCTTTCCTTCCATCC-3′; Reverse 5′-TAGCCAGTCCAGAGGTGAG-3′; FAS: Forward 5′-CCCTCCTACCTCTGGTTCTTACG-3′; Reverse 5’TCAGTCACTTGGGCATTAACACTTT-3′; FASL: Forward 5′-CCTGAAAAAAAGGAGCTGAGGAA-3′; Reverse 5′-GGCATGGACCTTGAGTTGGA-3′; GAPDH: Forward 5′-CAAGGCTGTGGGCAAGGT-3′; Reverse 5′-GGAAGGCCATGCCAGTGA-3:

Primers were designed at exon-exon junctions using Primer express 2.0 (Applied Biosystems). Target expression level was performed as previously described [19] using GAPDH as housekeeping gene. All the experiments were performed in triplicate. Data are expressed as the mean ± SD.

### Western blot

Protein extracts were prepared as previously described [20]. For each lane, 20 μg of total cell lysates were separated in 4–15% Tris–glycine gels (Bio-Rad Laboratories, Inc., CA, USA) at 100 V. Proteins were then transferred to PVDF membranes (Biorad Laboratories, Inc., CA, USA), probed with the specific primary antibodies, followed by secondary antibodies conjugated with horseradish peroxidase according manufacturer’s indications. Primary antibodies used for western blot include p53 (Cell Signaling, #2524) PARP (Cell Signaling, #9542), BCL-2 (Abcam, ab182858), BAX (Santa Cruz Biotechnology, sc-493), FAS (Abcam, ab133619) and Cytochrome *c* (Abcam, ab133504). β-Actin (Cell Signaling, #3700) was used as loading control. All the antibodies were used at working concentration indicated by manufacturers. Protein bands were detected by Clarity western ECL (Bio-Rad Laboratories, Inc., CA, USA) and quantified with ImageJ software. All the experiments were performed in triplicate. Data are expressed as the mean ± SD.

### Cytochrome *c* release

To determine the cytochrome *c* release from mitochondria to cytosol, cytosolic fractions were isolated resuspending 1 × 10^6^ cells in 100 μl of ice-cold plasma membrane permeabilization buffer (200 μg/ml digitonin, 80 mM KCl in PBS). After 5 min incubation on ice, lysates were centrifuged at 800 X g for 5 min at 4 °C, and the supernatants (cytosolic fraction) were then collected. Protein fractions were separated, transferred and probed with and Cytochrome *c* primary antibodies as described in the previous section.

### Mesothelioma xenograft tumor model

Xenograft mouse model of MM was induced by dorsal injection of human mesothelioma cells (MSTO-211H) as previously described [3].

Male CD1 Nude mice were purchased from Charles River Laboratories *Italia (Calco, Italy)* and housed in the animal Facility IRRC-Neuromed in accordance with protocols approved by the IRRC-Neuromed Animal Care Review Board and by Ministry of Health. In vivo experiments were conducted according to EU directive 2010/63/EU for animal experiments.

MSTO-211H cells (2.5 × 10^6^) were suspended in 0.2 ml serum-free DMEM medium and inoculated subcutaneously (s.c.) in the right flank of each mouse aged 5 weeks. 10 days after inoculation, when the tumours became visible, 8 mice for each experimental group were randomly assigned into control (DMSO) or treated groups (CBP or cisplatin) and treatments were administered by intra-peritoneal injection (i.p.).

To analyse anticancer properties of CBP, mice were daily treated with CBP (40 mg/kg) for 4 weeks or with Cisplatin (3.3 mg/kg) only for the first 3 days. Mice treated with vehicle alone (DMSO, daily for 4 weeks) were used as control.

Each tumor was measured weekly using a calliper; tumour’s size was assessed by using the formula: (long axis × short axis × short axis)/2.

The mice were sacrificed after 4-week treatments.

### Histology and immunohistochemistry

For histology, staining with hematoxylin/eosin and hematoxylin/Van Gieson were used. For immunohistochemistry, tissue sections were heated twice in a microwave oven for 5 min each at 700 W in citrate buffer (pH 6) and then processed with the standard streptavidin-biotin-immunoperoxidase method (DAKO Universal Kit, DAKO Corp., Carpinteria, CA, USA). Mouse monoclonal anti-human Ki67 (MIB-1 clone) and anti-CD31 (M0823 clone) antibodies from DAKO were used at a 1:100 dilution. Diaminobenzidine was used as the final chromogen, and hematoxylin as the nuclear counterstain. Negative control experiments for each tissue section were performed in the absence of the primary antibody. Positive controls, included in each experiment, consisted of tissue previously shown to express the antigen of interest. Two observers (S.C and A.B.), blinded to treatment conditions, evaluated the staining pattern of the proteins separately and quantitated the protein expression in each specimen by scanning the entire section and estimating the number of vessels or positive cells at the high-power-field 10 × 20. The level of concordance, expressed as the percentage of agreement between the observers, was 95%. In the remaining specimens, the score was obtained after collegial revision and agreement. The U-Mann Whitney test was used to assess relationship between ordinal data. Two-tailed *p*-value was considered significant when ≤0.05.

### TUNEL assay

TUNEL reaction was performed using the peroxidase-based Apoptag kit (Oncor, Gaithersburg, MD, USA). The experiment was repeated on at least two different sections for each specimen. Fifty random fields (250X) per section were analysed (6 mm^2^). The level of concordance, expressed as the percentage of agreement between the two observers (SC and AB), was 100%. The U-Mann Whitney test was used to assess relationship between ordinal data. Two-tailed *p*-value was considered significant when ≤0.05.

### Statistical analysis

Analysis was performed using Graph Pad Prism 6.0 (GraphPad Software, San Diego, CA, USA). Significance was evaluated using One-Way ANOVA with Bonferroni post hoc test for multiple comparisons or a Student’s *t*-test. p-value ≤0.05 was considered statistically significant.

## Results

### CBP determines growth inhibition in mesothelioma cells

We have previously reported that curcumin (20 μM) only when complexed with cyclodextrin can entry into cells determining cell viability decrease in mesothelioma cells [21].

**Fig. 1:**
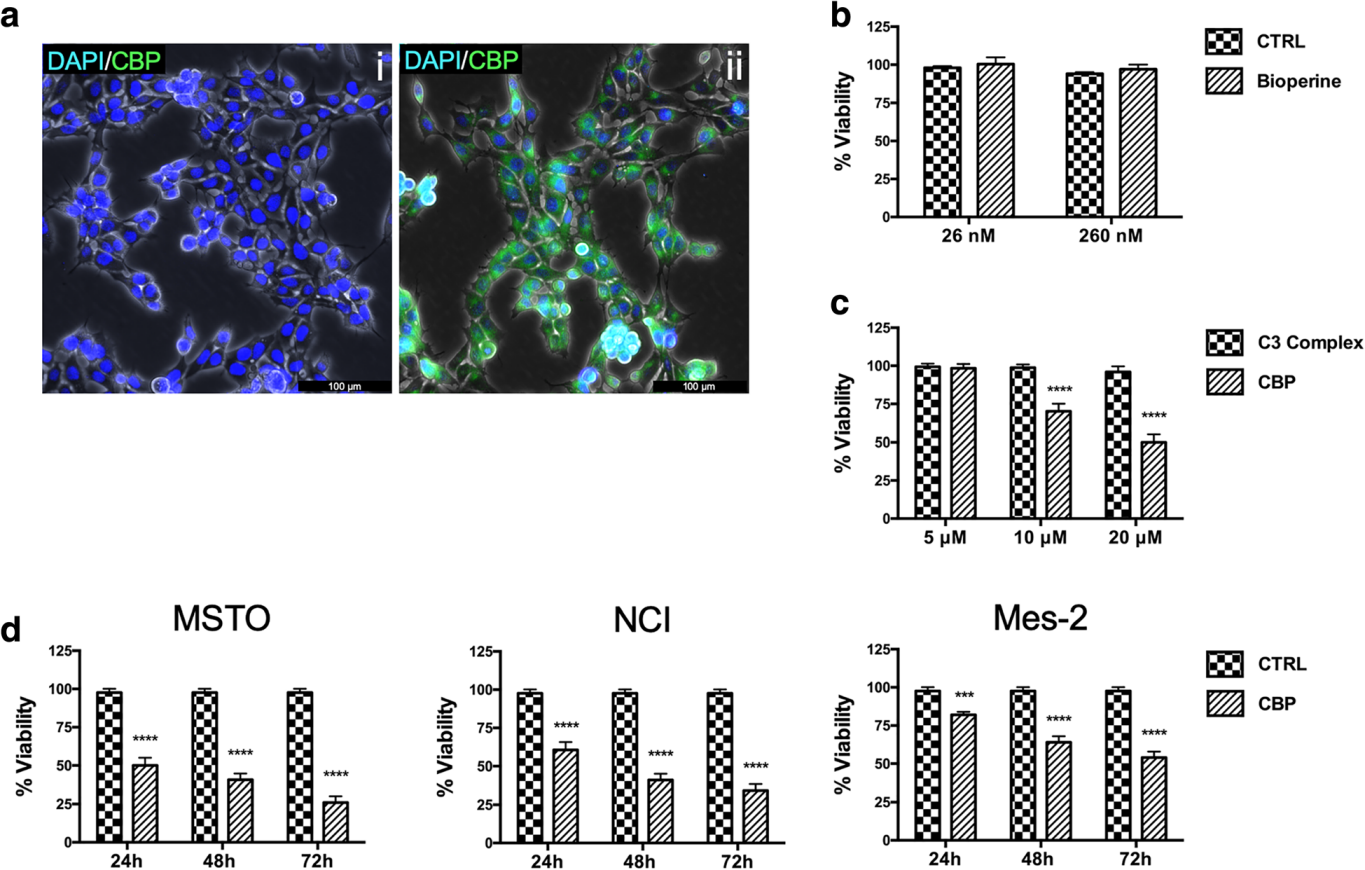
CBP uptake and effects on cell viability in mesothelioma cells. **a**) Fluorescence microscope analysis of MSTO cells incubated with DAPI (i) or with DAPI/CBP (ii) for 10′. Cellular uptake was analysed by curcumin intrinsic autofluorescence using a GFP filter. DAPI was used to counterstain nuclei. (**b**) Bioperine alone does not affect MSTO cell viability even when used 10X concentrated respect to the amount of BP present in CBP used for the experiments. **c**) Curcumin and Bioperine combined treatment increases C3 Complex bioactivity determining a cell viability decrease in dose dependent manner. d) Time-course measurement of cell viability decrease in different mesothelioma cell lines treated with 20 μM CBP for the indicated time. Cell viability was determined by crystal violet assay and cell viabilities are depicted as percentages. CTRL: untreated cells used as control. The indicators represent the average ± SD of independent experiments (*n* = 3). Statistically significant difference compared to untreated cells: ***p ≤ 0.001, ****p ≤ 0.0001. CRTL: untreated cells

To examine the C3 bioavailability, we first analysed whether C3 was able to penetrate MSTO-221H (MSTO) cells using the same concentration. Figure 1 a shows that C3 fluorescence is widely distributed in the cells, thus indicating that this complex is able to enter into cell through the plasma membrane.

Then, in order to investigate whether BP could enhance C3 bioactivity, we treated MSTO with different concentrations of C3 alone or combined with Bioperine. CBP resulted more effective than C3 complex alone (Fig. 1 c). In addition, Bioperine alone at same concentration present in CBP did not affect cell viability (Fig. 1 b). As shown in Fig. 1 c, CBP at 20 μM is able to determine a growth inhibition increase up to 40% compared to C3 alone, indicating that BP strongly determines a C3 bioavailability enhancement. Thus, we chose to use this concentration for the subsequent experiments.

To study the CBP in vitro activity we analysed the cell viability in a time course assay. The results indicated that CBP affects cell viability in a time dependent manner both in MSTO, and in NCI-H2452 (NCI) and in Ist-Mes-2 (Mes-2 cells) (Fig. 1 d).

Subsequently, cell proliferation, cell migration and invasive ability were analysed on all three cell lines in order to evaluate the anticancer potential CBP. The results clearly showed a complete growth inhibition and the loss of self-renewal ability and proliferative potential with a failure in colony formation.

**Fig. 2:**
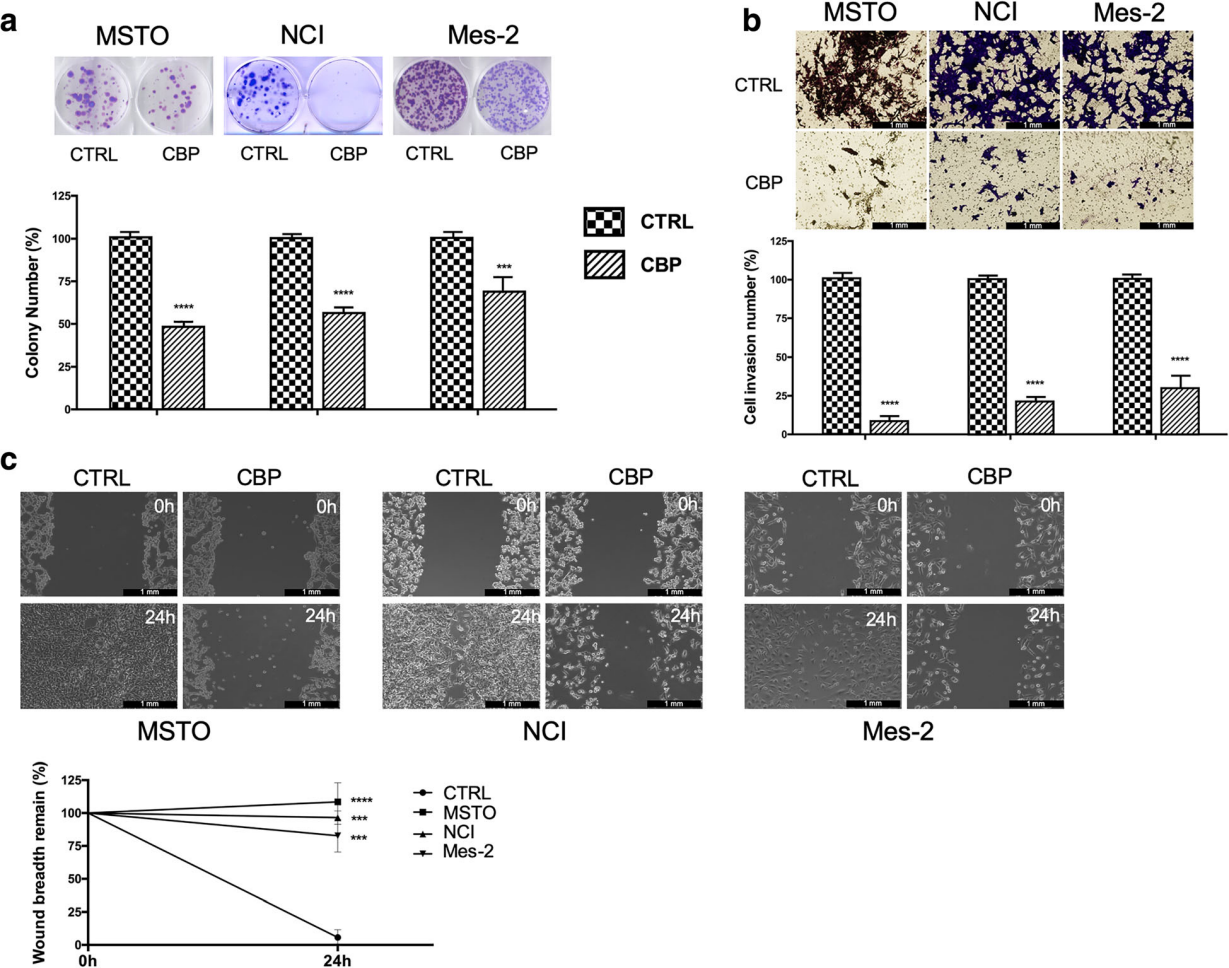
CBP impairs mesothelioma tumorigenic properties. **a**) The effect of CPB on colony forming ability of MM cell lines was analysed after 7 days of culture post CPB treatment. For quantification, colonies with at least 50 cells were considered**. b**) Invasive capability of human MM cells was determined using transwell invasion assay with Matrigel after 24 h CBP treatment as described in “Methods”. For each cell line five random fields in each well were counted under a microscope. Bright field images magnification: × 10. Representative micrographs shown in **a**) and **b**) were obtained after crystal violet staining. Histograms report average of colony or cell numbers respectively (% of control). c) Cell migration activity of mesothelioma cell lines was examined by wound healing assay. The wound closure rate was measured by detecting the closure distance after 24 h. Representative phase contrast images at 0 and at 24 h after CBP treatment (up) and quantification of the distance (down) are shown. Magnification of the upper panels: × 10. The indicators represent the average ± SD of independent experiments (n = 3). Statistically significant difference compared to untreated cells: **p ≤ 0.01, ***p ≤ 0.001, ****p ≤ 0.0001. CTRL: untreated cells

CBP was able to impair self-renewal ability and long-term proliferative potential in all the MM cell lines analysed. In fact, colony formation was almost completely inhibited after 24-h treatment with CBP (Fig. 2 a). Invasion abilities also resulted significantly reduced after CBP treatment (Fig. 2 b). Moreover, wound-healing assay showed that CBP significantly inhibited cell migration capacity in a time-dependent manner both in MSTO, and in NCI and in Mes-2 cells. In particular, the wound gap was about 100% after 24 h. (Fig. 2 c).

Taken together, these results indicated that CBP strongly reduces tumorigenic properties of MM cells, probably by affecting molecular pathways not involved in the response to the combined treatment with piroxicam and cisplatin since the effects are evident even in cells does not responding to this treatment [20].

### CBP induces apoptosis in mesothelioma cells

**Fig. 3:**
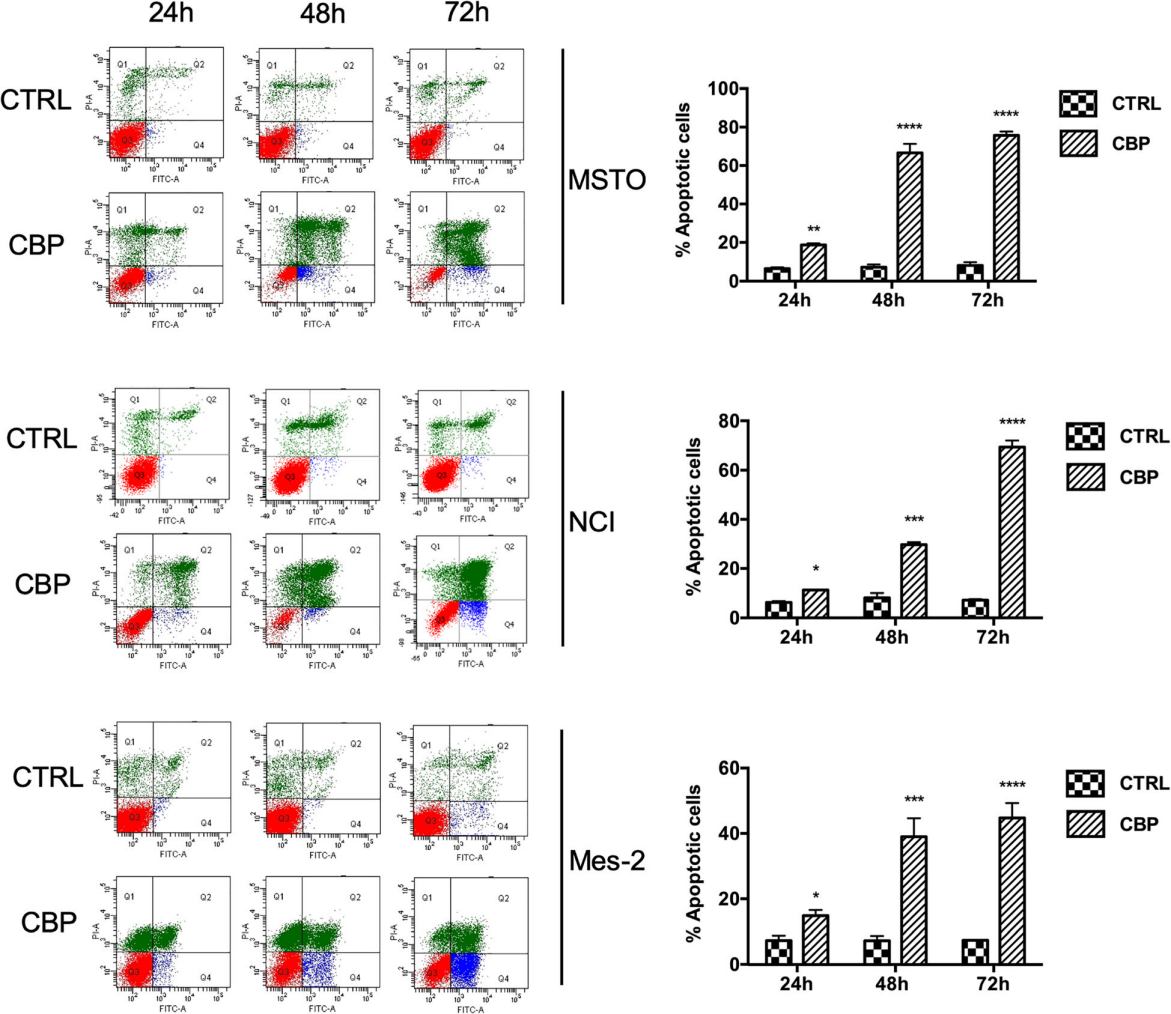
CBP treatments induce apoptosis in mesothelioma cells. FACS analysis by Annexin V-FITC/PI double staining assay for the detection of apoptosis in MSTO, NCI and Mes-2 cells treated with 20 μM CPB for 24, 48 and 72 h. Q2 and Q4: early and late apoptotic cells; Q1: necrotic fraction; Q3: live cells. Histograms on the right report a data summary of the apoptotic index. Bars represent the average ± SD of independent experiments (n = 3). Statistically significant difference compared to untreated cells: *p ≤ 0.05, **p ≤ 0.01, ***p ≤ 0.001, ****p ≤ 0.0001. CTRL: untreated cells

To understand whether cell viability impairment was due to apoptosis, we analysed the cell cycle perturbation and the downstream signalling triggered by CBP. MSTO, NCI and Mes-2 cells were treated with CBP for 24, 48 and 72 h and untreated cells were used as control. Apoptosis was investigated by flow cytometry analysis with AnnexinV-FITC/PI. The results clearly indicate that CBP determines a growing apoptotic induction during the time, reaching about 75% at 72 h (Fig. 3). On the contrary, in the untreated cells we did not detect any apoptotic increase over the time. We observed slight modifications in apoptosis among the different mesothelioma cell lines, with MSTO showing the highest sensitivity. More interestingly, CBP is effective also in Mes-2 cells that do not undergo to apoptosis after piroxicam and cisplatin treatment [17].

### CBP activates intrinsic apoptosis in mesothelioma cells

To investigate the molecular signalling pathway involved in the CBP-apoptotic cell death, we analysed if CBP was able to modulate p53 protein level and other apoptotic markers such as cleaved PARP, BAX and BCL2 FAS and FASL. Since untreated cells did not show modification of p53 levels or presence of PARP cleavage over the time (Additional file 1: Figure S1), we used as control samples proteins from untreated cells after 72 h culture that is the time where we observed the maximum CBP effect.

**Fig. 4:**
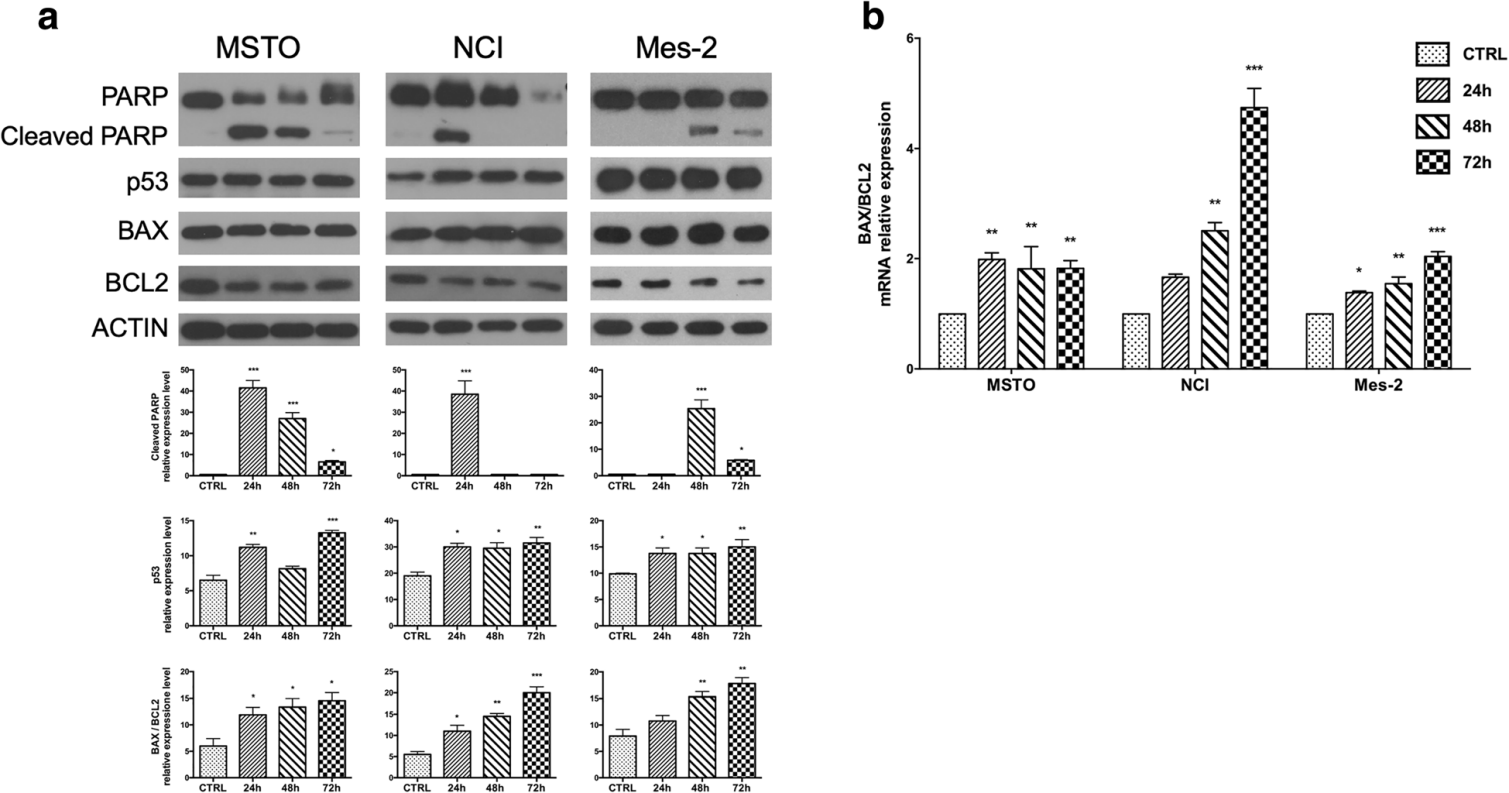
CBP induces intrinsic apoptosis. **a**) Protein expression levels of p53 and apoptotic markers in MM cell lines after 20 μM CPB for 24, 48 and 72 h treatments analysed by Western blot analysis. Histograms report the expression of p53, cleaved PARP and Bax/Bcl2 ratio normalized expression. β-Actin was used as loading control. **b**) Quantitative analysis of mRNA expression levels of *Bax* and *Bcl-2* in mesothelioma cell lines after 24, 48 and 72 h of CBP treatment. The bars represent ± the average ± SD of independent experiments (n = 3). Statistically significant difference compared to untreated cells: **p ≤ 0.01, ***p ≤ 0.005. CTRL: untreated cells after 72 h culture

Protein analysis shows an increase of the p53 level after CBP treatment in all MM cell lines, with MSTO showing the highest increase (Fig. 4 a). In addition, the presence of PARP cleavage confirmed activation of apoptosis.

Then we focused our attention on the molecular effectors involved in the activation of the *intrinsic* [22] or the *extrinsic* [23] apoptotic pathway, by analysing the expression of key molecules responsible for the two apoptotic pathways: BAX, BCL-2 and FAS, FASL respectively.

The analysis of BAX and BCL-2 protein expression indicated that CBP induced apoptosis through intrinsic pathway (Fig. 4 a)*.* Expression analysis by q-PCR also confirmed an increased ratio *BAX/Bcl-2* supporting the activation of intrinsic pathway (Fig. 4 b). According to these results we did not observe changes in the expression levels of FAS protein and gene in MSTO and NCI cells, while Mes-2 cells do not express FAS protein and mRNA (Additional file 2: Figure S2). On the contrary, we did not detect any expression of *FASL* genes in MM cells [24].

Finally, to further investigate the occurrence of intrinsic apoptotic pathway, we examined the cytochrome *c* release from mitochondria following CBP treatments. As shown in Additional file 2: Figure S2, CBP induced cytochrome *c* release in MM cell lines.

These results indicated that CPB in human MM induces apoptosis through intrinsic pathway.

### CBP shows anticancer activity in mesothelioma xenografts in mice

To verify the cellular and molecular in vitro results and analyse whether CBP may have beneficial effects in MM, we performed in vivo experiments using a xenograft mouse model of MM induced by dorsal injection of human mesothelioma cells (MSTO-211H).

**Fig. 5:**
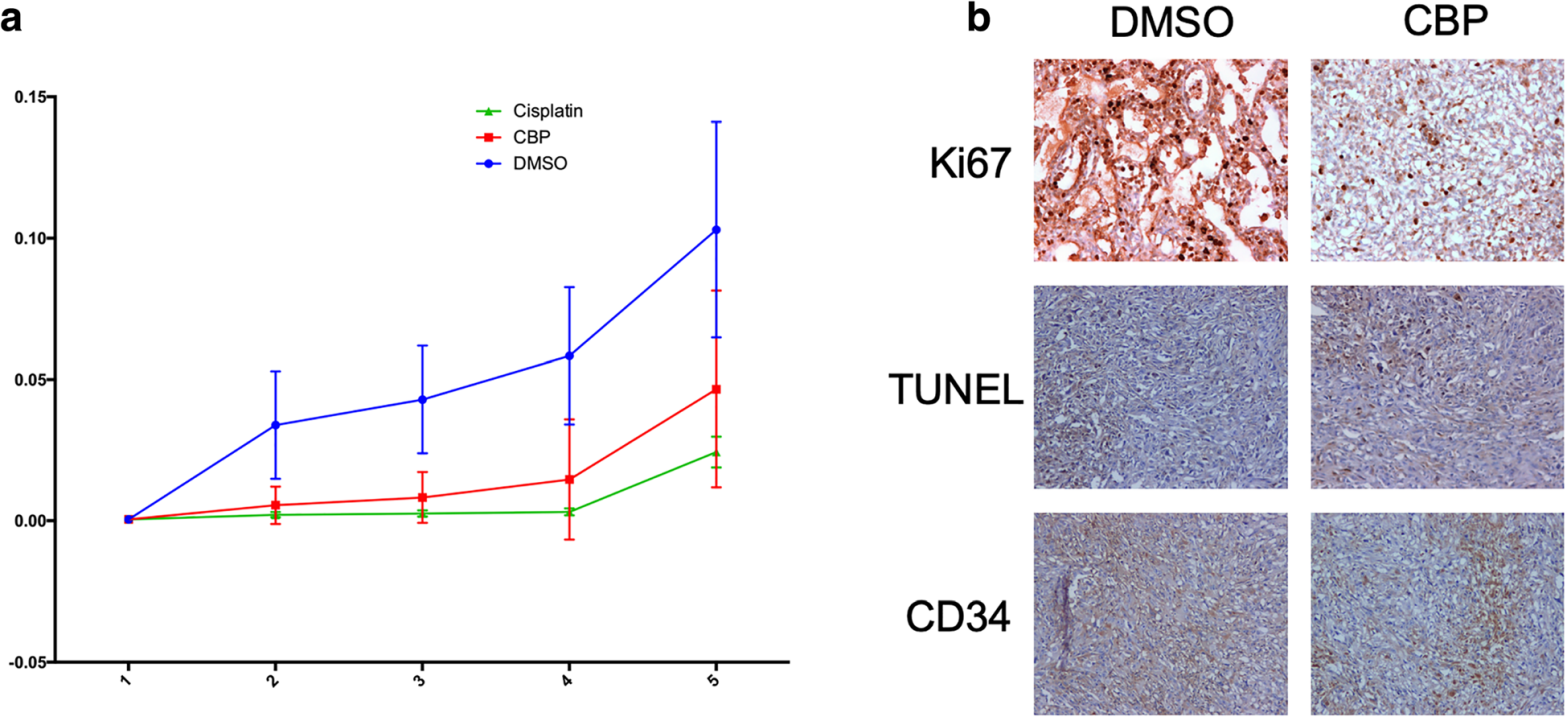
In vivo anticancer properties of CBP. **a**) Antitumor effect of CBP in a xenograft model of MM. The tumor growth curves show that CBP was able to significantly delay the growth of the ectopic tumors as compared to the controls (*p*-value = 0.0013), but with lower efficacy, compared to cisplatin (p-value = 0.0011). **b**) Effects of CBP on cell proliferation apoptosis and angiogenesis as compared to the control, as determined by Ki67, TUNEL (asterisks identify apoptotic cells) and CD34 staining (original magnification × 10). CBP treatment significantly decreased cell proliferation (25% vs 10%; p-value = 0.008), increased apoptosis (8% vs 2%; p-value = 0.0005) and decreased angiogenesis (5 ± 2 vs 10 ± 4; p-value = 0.0006)

In these experiments we analysed the effects of CBP and cisplatin (adopted as a positive control) treatments on tumour growth in comparison with treatment with vehicle (DMSO, control group). The results showed that daily administration of CBP (40 mg/kg) was able to significantly delay the growth of the ectopic tumours as compared to the controls (*p*-value = 0.0013), even if the efficacy of the treatment was lower in comparison with cisplatin (p-value = 0.0011) (Fig. 5 a). Histopathology analysis of ectopic tumours showed that treatment with CBP or cisplatin caused partial substitution of the tumor tissue by calcified and necrotic tissue. Tumor tissue from control samples displayed high proliferation index, as evaluated by Ki67 nuclear expression, low apoptotic index, as evaluated by TUNEL assay and high neo-angiogenesis, as detected by CD34 expression, as compared to CBP treatment (Fig. 5 b). In detail, Ki-67 expression was 25% in the controls vs. 10% in the treated tumours (p-value = 0.008), TUNEL score was 2% in the controls vs. 8% in the treated tumours (p-value = 0.0005), while the number of vessels in the control animals was 10 ± 4 and 5 ± 2 in the treated tumours (p-value = 0.0006). Interestingly, cisplatin treatment caused a similar effect on cell proliferation and angiogenesis as compared to CBP regimen, while the number of apoptotic cells was significantly higher with a TUNEL score higher than 10% (data not shown). The same CPB regimen was performed in nude mice not injected with MM cells to confirm the non-toxicity of this treatment (data not shown).

## Discussion

A major limitation of the conventional treatments for cancer is related to serious side effects that chemotherapeutic drugs may cause and to the development of cancer resistance. Some advances in cancer therapies have been currently reached thanks to the introduction of alternative approaches, including the use of several phytochemicals, which are able to significantly reduce tumor progression and to improve healing and survival [5].

MM is a rare and aggressive tumor developing from the mesothelial surface of the pleural space. Clinical signs are most of the time late and unspecific. The diagnosis is difficult with routine imaging procedures and usually it is based on immunohistochemistry of pleural biopsies. Until now neither surgical nor radiation treatments and conventional chemotherapy result in favorable prognosis for MM patients.

The objective of our study was to examine the anticancer activity of CBP in mesothelioma by in vitro and in vivo analysis.

The rationale for examining the potential efficacy of CBP as a MM chemopreventive agent was based on several lines of evidence. First, recent data suggest a robust anti-cancer efficacy of curcumin [4]. Second, some studies indicated that curcumin efficacy is tightly linked to its bioavailability [13, 14] and several approaches including complexation in nanoparticles [21] or phospholipid complexes [25] have been tested. Third, piperine significantly enhances curcumin bioavailability by inhibition of its glucuronidation processes [15, 16], and by increasing its molecular uptake in the intestine, since it reduces the intestinal transit [26]. Fourth, few investigations have demonstrated a specific anti-cancer activity on MM for curcumin reporting the in vivo anticancer effect of curcumin in mesothelioma mouse or rat allograft models [27–30].

In this work we analysed the anticancer activity of curcumin in MM using a specific formulation: C3 Complex®. This is a standardised extract from dried roots and rhizomes of *Curcuma longa* containing up to 95% of three different curcuminoids that constitute the active components: curcumin (79,97%), demethoxycurcumin (17,62%) and bisdemethoxycurcumin (2,41%). C3 Complex® was used in combination (1:100) with Bioperine®.

As a first line strategy, we performed an in vitro study to evaluate the effects of C3 Complex® in three different MM cell lines: MSTO, NCI, and Mes-2. The results showed that CBP compared to C3 alone greatly impaired cell viability in MM cells in a time dependent manner. Furthermore, CBP strongly reduced tumorigenic properties in MM by impairing cellular self-renewal ability, proliferative cell rate and cell migration. We also evidenced that CBP determines cell death through a time-dependent apoptotic induction in all the MM cell lines analysed. Subsequent analysis of the expression of key signalling molecules involved in apoptosis clearly indicated that CBP through p53 pathway activates intrinsic apoptotic pathway, since we detected an increase of the BAX/BCL-2 ratio at both protein and gene level and cyctochrome *c* release in all mesothelioma cell lines analysed. Indeed, unbalance between BCL-2 and BAX favours BAX activity. BAX induces mitochondrial membrane permeabilization and the subsequent cytochrome *c* release into the cytoplasm of MM cells. This determines the onset of apoptosis that culminates in caspases machinery activation and cleavage of PARP (poly(ADP-ribose) polymerase) protein [31].

To evaluate whether CBP could have chemopreventive effects also in vivo we generated a xenograft MM mouse model. The results indicated that daily administration of CBP up to four weeks delayed tumor growth even if to a lower extent when compared to cisplatin treatment. According to this observation, immunohistochemical analysis indicated that tumor samples from CBP-treated mice showed reduction of angiogenesis and apoptosis increase.

Different studies report that natural molecules can have anticancer properties sensitizing cancer cells to standard therapy [32]. Also curcumin has been reported to act in vivo synergizing drug effects, thus enhancing the global efficacy: examples are 5-fluorouracil or FOLFOX in colorectal cancer [33–35] or cisplatin in head and neck squamous cell carcinoma and ovarian cancer [36, 37]. Our results describe for the first time the efficacy of CBP in human mesothelioma xenograft mouse model. Although numerous studies describe the anti-cancer or the adjuvant effects of CBP [38, 39] and several clinical trials have been started [40, 41] none of them refers to MM. To our knowledge, this is the first study analysing in vitro and in vivo the efficacy of CBP on MM.

It is important to underline that in our experimental conditions CBP was ineffective in controlling bulky neoplasms. Nevertheless, by adopting this therapeutic scheme in a dose intensity protocol, it might be possible to achieve extended tumor control, especially considering that this approach could be associated to aggressive pleurectomy. Furthermore, considering the ability of curcumin to act in vivo synergizing drug effects, it is also possible that inclusion of other drugs in the treatment regimen may help achieve an additional increase in the anti-tumor efficacy of this therapy.

## Conclusions

In conclusion, our findings reveal novel, previously unappreciated anti-cancer effects of CBP in a model of MM, whose current prognosis remains very poor. Further studies aimed at delineating the exact molecular mechanisms responsible of these effects are required, since they are propaedeutic for future randomized clinical trials aimed at the evaluation of CBP as a chemotherapy agent in MM. Our results suggest that implementation of the standard pharmacological therapies with novel compounds may pave the way to develop alternative and more effective es to MM.

## Additional files

Additional file 1: **Figure S1.** p53 and PARP are not modulated in untreated cells. Analysis of p53 (a) and PARP protein (b) expression in untreated MSTO cultured for 24, 48 and 72 h by Western blot. There is neither modulation of p53 nor presence of cleaved PARP over the time. In b) MSTO treated with 20 μM CPB for 24 h were loaded as control of PARP cleavage. Histograms report the expression of p53, cleaved PARP normalized expression. β-Actin was used as loading control. The bars represent ± the average ± SD of independent experiments (*n* = 3). Statistically significant difference compared to untreated cells: ***p ≤ 0.001; ****p ≤ 0.0001. CTRL: untreated cells. (PNG 371 kb)

Additional file 2: **Figure. S2.** CBP does not induce death receptors but induces cytochrome *c* in mesothelioma cells. Protein (a) and gene (b) expression levels of FAS in MM cell lines after 20 μM CPB for 72 h analysed by Western blot and qPCR. FAS is not modulated in MSTO and NCI cells, while no protein and gene expression was detected in Mes-2 cells. c) Western blot analysis showing cytosolic release of cytochrome *c* in MM cell lines after 20 μM CBP treatment at 72 h. Histograms report the expression of FAS or cytochrome *c* normalized expression. In western blot experiments β-Actin was used as loading control. The bars represent ± the average ± SD of independent experiments (n = 3). Statistically significant difference compared to untreated cells: ****p ≤ 0.0001. CTRL: untreated cells after 72 h culture. (PNG 750 kb)

## Data Availability

All data generated or analysed during this study are included in this published article. The original submitted files for images are available from the corresponding author upon request.

## Abbreviations

BAX: Bcl-2-associated X protein
BCL-2: B-cell lymphoma 2
CBP: C3 complex® and Bioperine®
FACS: Fluorescence activated cell sorting
FASL: Fas ligand
MM: malignant mesothelioma
PARP: (poly(ADP-ribose) polymerase) protein
