# Supplementary figures and images for: Curcumin C3 complex®/Bioperine® has antineoplastic activity in mesothelioma: an in vitro and in vivo analysis
Source: J Exp Clin Cancer Res. 2019 Aug 16;38:360. doi: 10.1186/s13046-019-1368-8 (PMC6698046; doi:10.1186/s13046-019-1368-8)

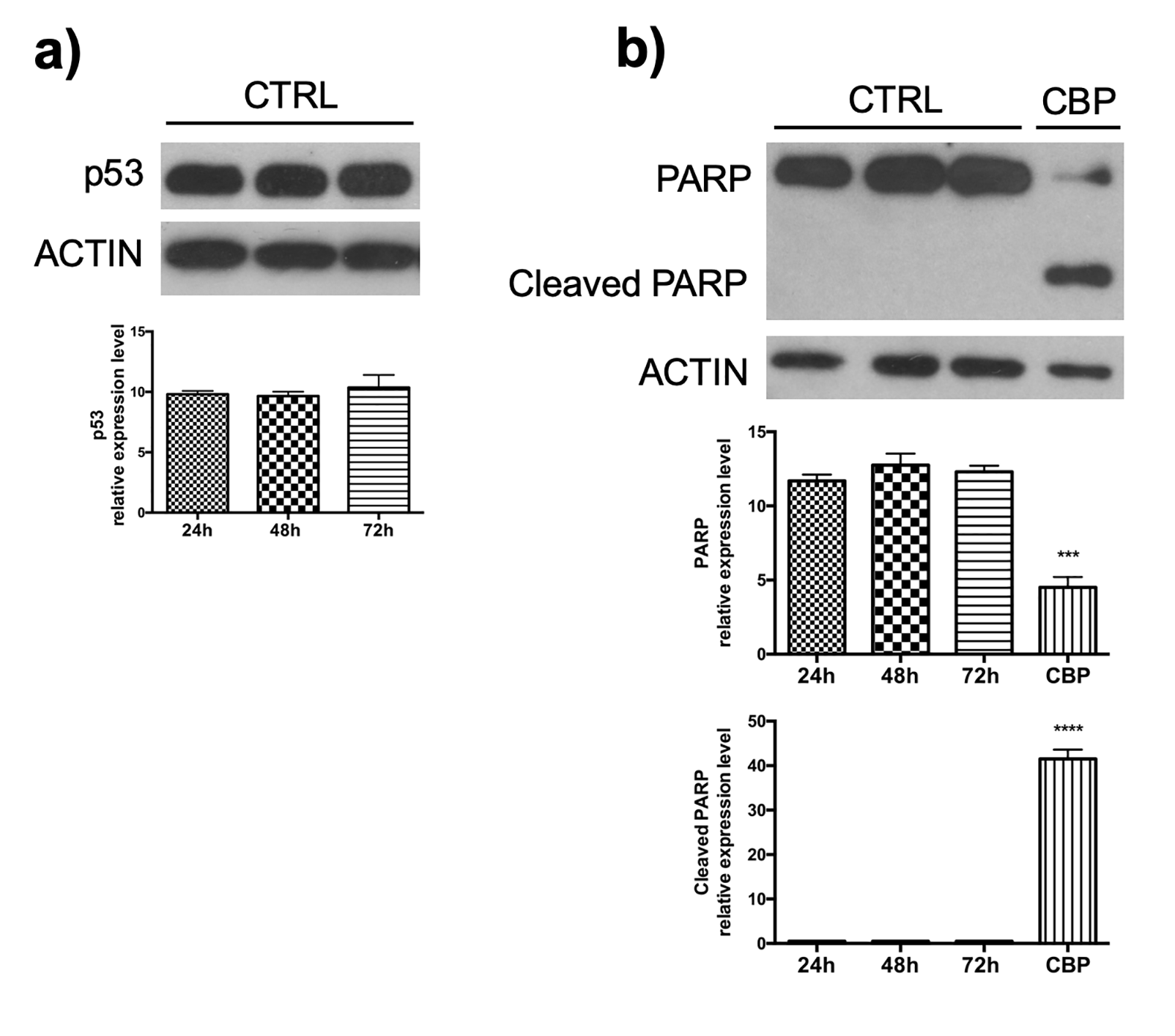

Supplement: Supplementary file 1 — Figure S1. p53 and PARP are not modulated in untreated cells. Analysis of p53 (a) and PARP protein (b) expression in untreated MSTO cultured for 24, 48 and 72 h by Western blot. There is neither modulation of p53 nor presence of cleaved PARP over the time. In b) MSTO treated with 20 μM CPB for 24 h were loaded as control of PARP cleavage. Histograms report the expression of p53, cleaved PARP normalized expression. β-Actin was used as loading control. The bars represent ± the average ± SD of independent experiments (n = 3). Statistically significant difference compared to untreated cells: ***p ≤ 0.001; ****p ≤ 0.0001. CTRL: untreated cells. (PNG 371 kb) [file 13046_2019_1368_MOESM1_ESM.png]

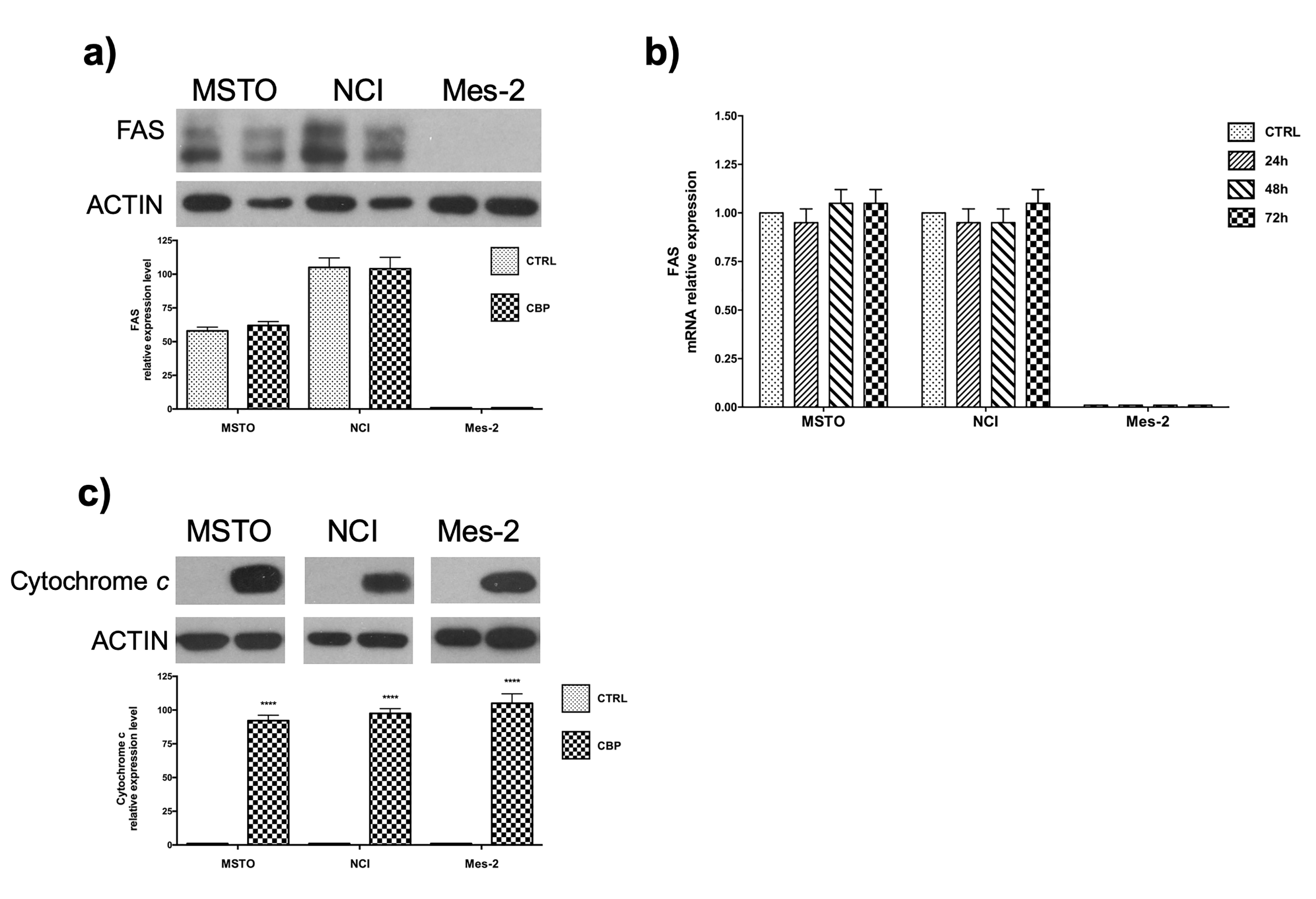

Supplement: Supplementary file 2 — Figure. S2. CBP does not induce death receptors but induces cytochrome c in mesothelioma cells. Protein (a) and gene (b) expression levels of FAS in MM cell lines after 20 μM CPB for 72 h analysed by Western blot and qPCR. FAS is not modulated in MSTO and NCI cells, while no protein and gene expression was detected in Mes-2 cells. c) Western blot analysis showing cytosolic release of cytochrome c in MM cell lines after 20 μM CBP treatment at 72 h. Histograms report the expression of FAS or cytochrome c normalized expression. In western blot experiments β-Actin was used as loading control. The bars represent ± the average ± SD of independent experiments (n = 3). Statistically significant difference compared to untreated cells: ****p ≤ 0.0001. CTRL: untreated cells after 72 h culture. (PNG 750 kb) [file 13046_2019_1368_MOESM2_ESM.png]
